# Signaling Pathway Puts the Break on Fat Cell Formation

**DOI:** 10.1100/tsw.2001.28

**Published:** 2001-05-01

**Authors:** Ormond A. MacDougald

**Affiliations:** Department of Physiology, University of Michigan Medical School, Ann Arbor 48109-0622, USA

**Keywords:** obesity, adipocye, fat, wnt, health

## Abstract

Obesity is approaching epidemic proportions in the western industrialized world, and is also becoming a major problem among young people in eastern and developing countries [1,2,3]. Unfortunately, excess fat or adipose tissue is associated with a wide array of health problems, including increased incidence of type II diabetes, cardiovascular disease, hypertension, sleep apnea, and skeletomuscular problems [4,5,6]. Obesity is the second leading cause of death from “unnecessary” causes in the U.S. (after smoking), and costs individuals and society billions of dollars worldwide to treat. Despite common wisdom that “one just needs to eat less and exercise more” and a multi-billion-dollar diet industry, epidemiological data indicate that the incidence of obesity will continue to rise. This alarming trend is, in part, due to the unprecedented availability of energy-dense foods and an increasingly sedentary lifestyle. These environmental factors may be complicated in some individuals by an unfavorable genetic predisposition. Pharmaceutical companies lead active research programs to identify drugs that target weight control centers in the body and which may help individuals control their weight; however, no satisfactory magic bullet to fight obesity has yet come through the pipeline [7,8].

A recent study published in Science [9] reveals a signaling pathway that actively represses the development of precursor cells into fat cells. The signaling pathway, initiated by secreted proteins called Wnts, acts to stop the induction of master regulatory proteins that control production of adipose genes. Thus, fat cell formation is stopped before it even gets started. This finding is particularly significant because, although much is known about the processes that stimulate fat cell formation, little information is known about the signals that inhibit the process [10,11,12,13,14].

Using established cell lines that turn into fat cells when cultured under the right conditions, these investigators used a number of methods to demonstrate that inappropriate activation of the Wnt signaling pathway blocks development of fat cells in culture. Similar results are also observed when the precursor cells are implanted subcutaneously in mice [9]. Thus, expression of Wnts is sufficient to block adipogenesis. The investigators also showed that inhibiting the intrinsic Wnt signal within precursor cells results in their differentiation into adipocytes in the absence of any exogenous inducing agents. The fact that blocking the Wnt signaling pathway results in spontaneous fat cell conversion indicates that these precursor cells are all primed and ready to turn into fat cells, and that a brake supplied by the Wnt pathway is all that is keeping them from becoming fat. A particularly surprising finding from these University of Michigan researchers is that the Wnt signaling pathway also acts to keep muscle precursor cells on the path to becoming muscle, and that disruption of this signaling pathway causes these muscle precursors to develop into fat cells [9]. This may provide an intriguing molecular mechanism for the common complaint that "my muscles have gone to flab."

Although this research provides molecular insight into the signaling pathways that regulate fat cell development, it is unlikely to provide a critical target for drug development. Since Wnts are used to direct the development of many cell types within the body [15,16,17], there could be significant side effects for a drug that regulates the Wnt signaling pathway. If a Wnt-mimicking drug could be targeted selectively to adipose tissue, its effect would be to reduce adipose mass over the long term as cells are turned over and not replaced. Because much of obesity occurs due to an increase in the fat stored per adipocyte rather than an increase in the number of adipocytes, this target is unlikely to be the quick-fix for obesity that people want. The best target sites for weight loss drugs remain in the brain centers that control appetite and whole body energy metabolism, both of which can play important causative roles in the development of obesity [7,8]. Although Wnts are not likely to be the magic bullet for weight loss, they do provide important mechanistic information on how the formation of adipocytes is controlled, and mutations within the Wnts signaling components could be part of the genetic predisposition of some individuals to put on the pounds.

| | | |
|---|---|---|
| Received: | February | 15, 2001 |
| Accepted: | March | 26, 2001 |
| Published: | May | 1, 2001 |
